# Antifungal Efficacy of Chlorhexidine Digluconate and Silver Nanoparticles Incorporated into Molloplast-B Silicone Soft Liner Against Candida albicans: An In Vitro Study

**DOI:** 10.4317/jced.63625

**Published:** 2026-03-30

**Authors:** Samira Soufiabadi, Mahsa Khodaparast, MohammadHossein Khodaparast, Sahar Khodaparast, Hoda Shabani Attar, Reihaneh Abedi

**Affiliations:** 1 DDS. Assistant Professor, Department of Prosthodontics. Faculty of Dentistry, Kerman University of Medical Sciences , Kerman, Iran; 2 DDS. Royal College of Dental Surgeon, Ontario, Canada; 3 School of Stomatology, Zhengzhou University, Zhengzhou, Henan, China; 4 MD. Faculty of Medicine, Kerman University of Medical Sciences, Iran; 5 DDS. Resident, Department of Prosthodontics. Kerman University of Medical Sciences, Iran

## Abstract

**Background:**

Denture stomatitis is a common inflammatory condition among complete denture wearers and is primarily associated with Candida albicans colonization on denture base and soft lining materials. Conventional antifungal therapies often provide only temporary benefits due to limited patient compliance and rapid microbial recolonization. Therefore, intrinsic antifungal modification of silicone-based soft liners may represent a more effective preventive strategy.

**Material and Methods:**

Standardized Molloplast-B silicone soft liner discs (6 mm × 1 mm; n = 10 per group) were modified with 1%, 2%, or 3% (w/w) chlorhexidine digluconate (CHX) or silver nanoparticles (AgNPs) using a solvent-assisted dissolution technique followed by heat polymerization. Unmodified Molloplast-B, solvent-processed Molloplast-B, pure CHX solution, and AgNP suspension served as control groups. Antifungal activity against Candida albicans ATCC 10231 was evaluated using an agar diffusion assay after 24-hour incubation at 37°C. Inhibition zones were measured by two blinded examiners. Data were analyzed using one-way ANOVA and Tukey's post-hoc test ( = 0.05).

**Results:**

All modified groups demonstrated significantly greater antifungal activity compared with the negative and solvent controls (P &lt; 0.001). CHX-modified specimens exhibited significantly larger inhibition zones than AgNP-modified specimens at all tested concentrations (P &lt; 0.001). The highest antifungal activity was observed in the 3% CHX group (22.6 ± 1.8 mm), followed by the 3% AgNP group (16.0 ± 1.1 mm). A clear dose-dependent increase in antifungal efficacy was observed for both antimicrobial agents.

**Conclusions:**

Incorporation of 3% chlorhexidine digluconate into Molloplast-B silicone soft liner resulted in superior short-term antifungal efficacy against Candida albicans compared with silver nanoparticles. This intrinsic modification approach may be a promising strategy for reducing fungal colonization and preventing denture stomatitis; however, further long-term and clinical studies are required.

## Introduction

Soft denture liners are commonly used in prosthodontics to improve patient comfort by cushioning atrophic alveolar ridges, distributing occlusal forces more evenly, and reducing mucosal trauma, particularly in elderly or systemically compromised patients (1 , 2). Silicone-based liners such as Molloplast-B are frequently selected for long-term clinical use due to their superior elasticity, durability, and resilience compared with acrylic-based materials (1). Despite their clinical advantages, soft denture liners are highly susceptible to microbial colonization, which significantly limits their longevity and clinical performance. Denture stomatitis is a prevalent inflammatory condition affecting approximately 40-70% of complete denture wearers and is especially common among institutionalized elderly individuals (3 - 5). Candida albicans is considered the primary etiologic agent of denture stomatitis, as it readily adheres to the porous and hydrophobic surfaces of silicone liners and forms resilient biofilms (6 , 7). The adhesion and proliferation of C. albicans on denture liners are influenced by several factors, including poor oral hygiene, continuous denture wearing, xerostomia, and systemic conditions such as diabetes and immunosuppression (8). Once established, fungal biofilms contribute not only to mucosal inflammation characterized by erythema and edema, but also to material degradation, discoloration, malodor, and the need for frequent relining or replacement of the prosthesis (9). Conventional management of denture stomatitis typically involves topical antifungal agents such as nystatin or miconazole. However, these treatments often provide only short-term relief due to limited penetration into biofilms, inadequate patient compliance, and rapid recolonization after treatment cessation (10 , 11). Systemic antifungal therapies may lead to adverse effects and the development of antifungal resistance, further emphasizing the need for alternative preventive approaches (12). Incorporation of antimicrobial agents directly into the polymer matrix of denture liners has been proposed as an effective strategy to achieve sustained antifungal activity (13). Chlorhexidine digluconate (CHX) is a broad-spectrum antimicrobial agent that disrupts fungal cell membranes, inhibits enzymatic activity, and interferes with biofilm formation (14). Silver nanoparticles (AgNPs) exert antifungal effects through multiple mechanisms, including the generation of reactive oxygen species, membrane destabilization, and interference with DNA replication, and have demonstrated acceptable biocompatibility at low concentrations (15 , 16). Previous studies have demonstrated antifungal efficacy of both CHX and AgNPs when incorporated into silicone-based materials, often at concentrations up to 3% (w/w) without inducing significant cytotoxic effects (17 , 18). However, direct comparisons of these two antimicrobial agents within the same heat-cured silicone soft liner system remain limited, and the optimal concentration for achieving effective antifungal activity in Molloplast-B has not been clearly established (19 , 20). Unlike previous investigations that primarily evaluated acrylic-based liners or single antimicrobial agents, the present study provides a direct head-to-head comparison of chlorhexidine digluconate and silver nanoparticles incorporated into heat-cured Molloplast-B silicone soft liner using a solvent-assisted processing technique. This approach enables uniform dispersion of antifungal agents within the polymer matrix and allows evaluation of dose-dependent antifungal efficacy under standardized experimental conditions. By focusing on Molloplast-B, a widely used long-term silicone liner, the present findings offer clinically relevant insights into intrinsic antifungal modification strategies for the prevention of denture stomatitis. Accordingly, the aim of this in vitro study was to evaluate and compare the antifungal efficacy of Molloplast-B silicone soft liner modified with different concentrations (1-3% w/w) of chlorhexidine digluconate or silver nanoparticles against Candida albicans using an agar diffusion assay.

## Materials and Methods

**Figure 1:**
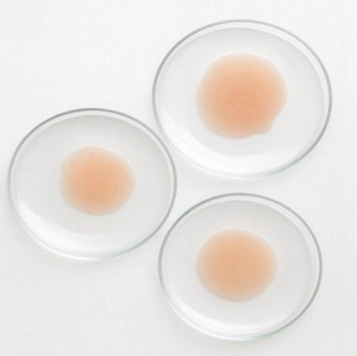
Molloplast-B mixtures post-chloroform evaporation.

This in vitro experimental study was conducted to evaluate the antifungal efficacy of modified Molloplast-B silicone soft denture liner against Candida albicans using an agar diffusion assay. As this was a laboratory-based in vitro investigation, formal ethical approval was not required. - Sample Preparation Molloplast-B silicone soft liner (Detax GmbH &amp; Co. KG, Ettlingen, Germany) was used as the base material. To facilitate uniform incorporation of antimicrobial agents, the material was dissolved in chloroform (Merck, Darmstadt, Germany) at a 1:1 weight ratio to obtain a homogeneous paste. Chlorhexidine digluconate (CHX) (20% aqueous solution; Sigma-Aldrich, St. Louis, MO, USA) or silver nanoparticles (AgNPs) (average particle size: 18 nm; concentration: 4000 ppm; Nanocid®, Nano Nasb Pars Co., Tehran, Iran) were added to the paste at concentrations of 1%, 2%, or 3% (w/w). The appearance of Molloplast-B mixtures after complete chloroform evaporation is illustrated in Fig. 1.

1

**Figure 2:**
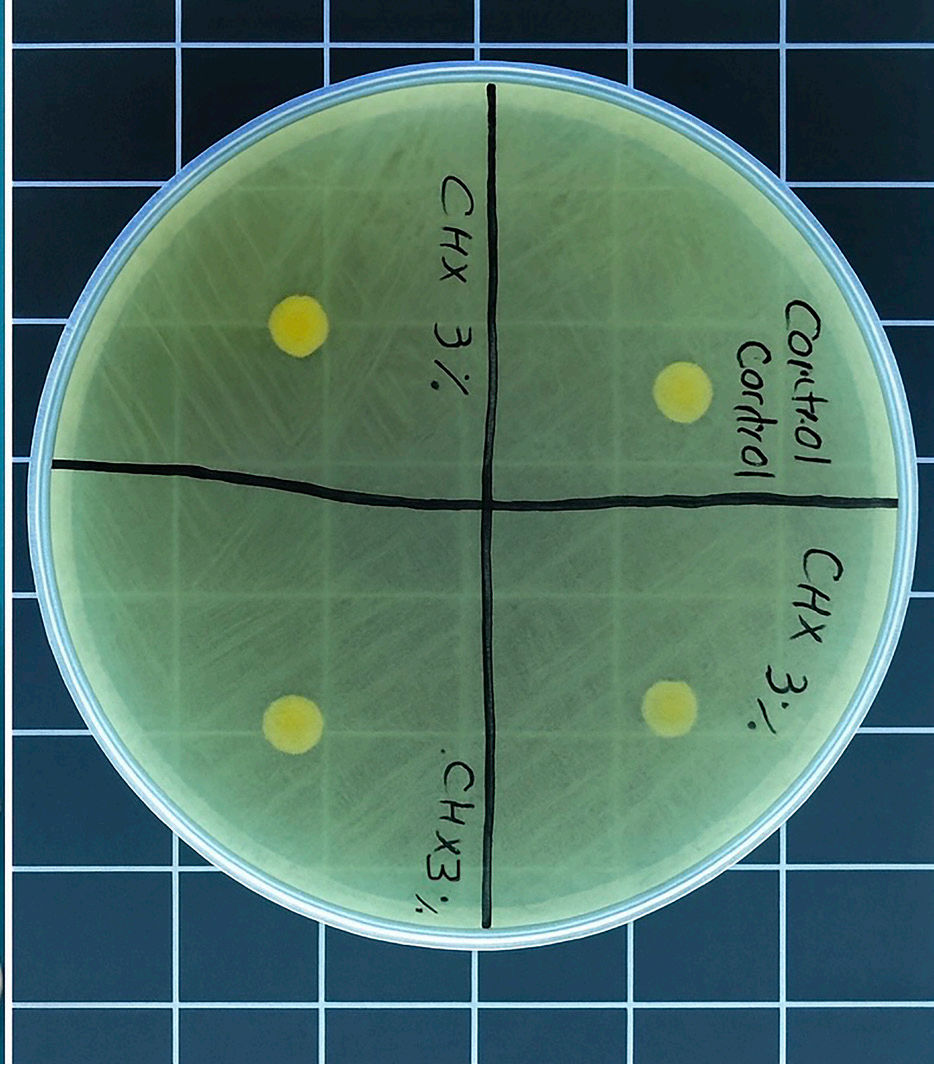
Representative agar plates with inhibition zones.

Each mixture was stirred at 300 rpm for 5.5 hours at room temperature (25°C) to ensure uniform dispersion of the antimicrobial agents. The solvent was then allowed to evaporate under a fume hood for 24 hours until a constant weight was achieved, confirming complete chloroform removal. The resulting material was packed into custom-made brass molds with standardized dimensions (6 mm diameter × 1 mm thickness) and heat polymerized at 100°C for 2 hours according to the manufacturer's instructions. After polymerization, the specimens were deflasked, trimmed, and polished using 600-grit silicon carbide abrasive paper to obtain uniform surfaces. Each specimen was examined under ×10 magnification to detect surface defects, and any defective samples were discarded and replaced. A total of ten specimens were prepared for each experimental group. - Control Groups Four control groups were included: unmodified Molloplast-B specimens (negative control), Molloplast-B specimens processed with chloroform only (solvent control), pure 20% chlorhexidine digluconate solution (positive control), and a 4000 ppm silver nanoparticle suspension (positive control). - Sterilization Procedure All specimens were sterilized using ethylene oxide gas at 55°C for 4 hours, followed by a 7-day aeration period to eliminate residual gas and prevent interference with antifungal activity. - Microbiological Procedures A standard strain of Candida albicans (ATCC 10231) was used in this study. The microorganism was revived on Sabouraud dextrose agar (SDA; Merck, Darmstadt, Germany) and incubated at 37°C for 48 hours. A single colony was then transferred to Sabouraud dextrose broth and incubated until the logarithmic growth phase was achieved. The fungal suspension was adjusted to 0.5 McFarland standard, corresponding to approximately 1.5 × 108 CFU/mL, using a spectrophotometer at a wavelength of 625 nm. - Agar Diffusion Assay For antifungal evaluation, 20 mL of molten SDA was poured into sterile Petri dishes and allowed to solidify. Each plate was inoculated with 100 µL of the standardized C. albicans suspension, which was evenly spread across the agar surface. The prepared specimens were then placed onto the agar plates under aseptic conditions. All plates were incubated at 37°C for 24 hours. Representative agar plates demonstrating inhibition zones around the specimens are shown in Fig. 2.

2

After incubation, the diameters of the inhibition zones surrounding each specimen were measured in millimeters using a digital caliper with 0.01 mm accuracy. Measurements were performed independently by two blinded examiners. Intra-examiner and inter-examiner reliability were assessed using intraclass correlation coefficients (ICC), which were 0.96 and 0.95, respectively. - Statistical Analysis Data distribution was assessed using the Kolmogorov-Smirnov test and confirmed to be normal (P &gt; 0.05). Differences in inhibition zone diameters among groups were analyzed using one-way analysis of variance (ANOVA). Pairwise comparisons were performed using Tukey's post-hoc test. Statistical analysis was conducted using SPSS software (version 26.0; IBM Corp., Armonk, NY, USA), with the level of significance set at = 0.05. Results were reported as mean ± standard deviation with corresponding 95% confidence intervals.

## Results

**Figure 3:**
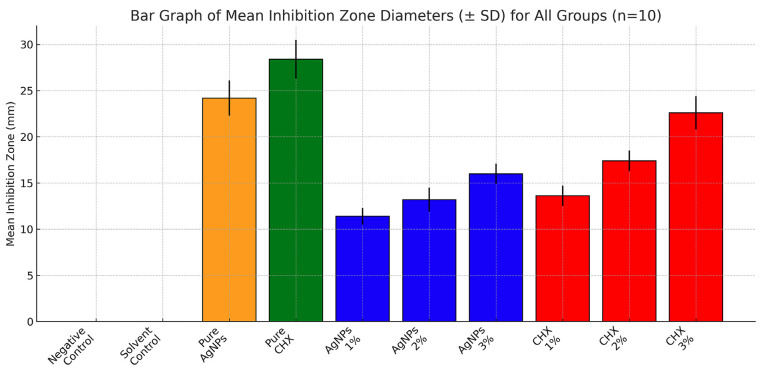
Bar graph of mean inhibition zone diameters (± SD) for all groups (n=10).

All experimental groups modified with chlorhexidine digluconate (CHX) or silver nanoparticles (AgNPs), as well as the positive control groups, demonstrated measurable antifungal activity against Candida albicans. In contrast, neither the unmodified Molloplast-B specimens nor the solvent-processed specimens exhibited any inhibition zones (0.0 ± 0.0 mm). Statistical analysis revealed a highly significant difference in inhibition zone diameters among the study groups (one-way ANOVA, F = 456.2, df = 9, P &lt; 0.001), with a large effect size (² = 0.92). Post-hoc comparisons using Tukey's test confirmed that all modified groups differed significantly from the negative and solvent controls (P &lt; 0.001). The pure chlorhexidine digluconate solution exhibited the largest mean inhibition zone (28.4 ± 2.1 mm; 95% CI: 26.9-29.9 mm), followed by the pure silver nanoparticle suspension (24.2 ± 1.9 mm; 95% CI: 22.8-25.6 mm). Among the modified Molloplast-B groups, a clear concentration-dependent increase in antifungal activity was observed for both antimicrobial agents. For CHX-modified specimens, mean inhibition zone diameters increased from 13.6 ± 1.1 mm (1% CHX; 95% CI: 12.8-14.4 mm) to 17.4 ± 1.1 mm (2% CHX; 95% CI: 16.6-18.2 mm), reaching a maximum of 22.6 ± 1.8 mm at 3% CHX (95% CI: 21.3-23.9 mm). Similarly, AgNP-modified specimens demonstrated inhibition zones of 11.4 ± 0.9 mm (1% AgNPs; 95% CI: 10.8-12.0 mm), 13.2 ± 1.3 mm (2% AgNPs; 95% CI: 12.3-14.1 mm), and 16.0 ± 1.1 mm (3% AgNPs; 95% CI: 15.2-16.8 mm). At each corresponding concentration level, CHX-modified groups exhibited significantly larger inhibition zones than AgNP-modified groups (P &lt; 0.001). A graphical comparison of mean inhibition zone diameters for all experimental and control groups is presented in Fig. 3, (Table 1).

3

Table 1

## Discussion

The present in vitro study evaluated and compared the antifungal efficacy of Molloplast-B silicone soft liner modified with chlorhexidine digluconate (CHX) or silver nanoparticles (AgNPs) against Candida albicans. The results demonstrated that incorporation of both antimicrobial agents significantly enhanced the antifungal properties of Molloplast-B compared with unmodified and solvent-processed controls. Notably, CHX-modified specimens consistently exhibited greater antifungal activity than AgNP-modified specimens at all tested concentrations. The superior performance of CHX may be attributed to its well-established mechanism of action, which involves disruption of fungal cell membranes, increased membrane permeability, and inhibition of enzymatic activity, ultimately leading to cell death. In addition, CHX exhibits favorable diffusion characteristics within agar-based media, which may partially explain the larger inhibition zones observed in comparison with AgNPs. Similar findings have been reported in previous studies evaluating CHX-modified silicone liners, where concentration-dependent antifungal effects were consistently observed (13 , 14 , 17). In contrast, AgNPs exert their antifungal activity primarily through the release of silver ions and the generation of reactive oxygen species, which disrupt cellular structures and interfere with DNA replication (15 , 16). However, when incorporated into hydrophobic silicone matrices, the release and diffusion of silver ions may be limited, potentially accounting for the smaller inhibition zones observed in the present study. Nonetheless, the dose-dependent increase in antifungal efficacy observed with AgNP-modified specimens suggests that silver nanoparticles remain a viable antimicrobial additive, particularly for long-term applications where sustained release may be advantageous. Previous studies published in the Journal of Clinical and Experimental Dentistry have emphasized the critical role of Candida albicans biofilm formation on denture base and lining materials in the pathogenesis of denture stomatitis, as well as the clinical importance of strategies aimed at reducing fungal adhesion and colonization (21). In agreement with these findings, the present study demonstrated that intrinsic antifungal modification of a silicone soft liner can effectively inhibit fungal growth, supporting the potential clinical value of this preventive approach. The solvent-assisted processing technique used in this study facilitated homogeneous dispersion of antimicrobial agents within the Molloplast-B matrix, which may have contributed to the consistent antifungal performance observed across specimens. Moreover, the use of heat-cured Molloplast-B, a widely utilized long-term silicone liner, enhances the clinical relevance of the findings, as results obtained with temporary or acrylic-based liners may not be directly translatable to long-term silicone materials (22 , 23). The novelty of this study lies in the direct head-to-head comparison of two commonly proposed antifungal agents within the same heat-cured silicone liner system under identical experimental conditions. Unlike previous investigations that evaluated these agents separately or in different materials, the present design allowed a clinically meaningful comparison of their relative antifungal performance rather than isolated efficacy assessment (24 , 25). Despite the promising results, several limitations should be acknowledged. This study was conducted under in vitro conditions and therefore did not account for complex oral environmental factors such as salivary flow, pH fluctuations, mechanical wear, or multispecies biofilm formation. Additionally, only a standard laboratory strain of C. albicans was evaluated, and the antifungal activity was assessed after a short incubation period of 24 hours. Future studies should investigate long-term antifungal release, color stability, mechanical properties, cytotoxicity, and the efficacy of these modified liners against clinical isolates and mixed microbial biofilms under simulated oral conditions (26 - 30).

## Conclusions

Within the limitations of this in vitro study, incorporation of antimicrobial agents into Molloplast-B silicone soft denture liner significantly enhanced its antifungal activity against Candida albicans. Among the evaluated modifications, chlorhexidine digluconate demonstrated superior antifungal efficacy compared with silver nanoparticles at all tested concentrations. The highest antifungal effect was observed with 3% chlorhexidine digluconate incorporation, indicating a clear concentration-dependent response and suggesting that this formulation may be particularly effective for reducing fungal colonization on silicone soft liners. Although silver nanoparticles also exhibited dose-dependent antifungal activity, their overall efficacy was lower than that of chlorhexidine digluconate under the conditions of this study. These findings support the potential clinical application of intrinsic antifungal modification of silicone soft liners as a preventive strategy for denture stomatitis. However, further investigations are required to evaluate long-term antifungal release, mechanical and esthetic properties, biocompatibility, and clinical performance under simulated oral conditions and in vivo settings.

## Figures and Tables

**Table 1:** Mean inhibition zone diameters (mm ± SD) against Candida albicans ATCC 10231 after 24-hour incubation (n=10/group). All modified groups and positive controls showed significantly larger zones than negative/solvent controls (P<0.001).

Group	Mean ± SD	95% CI	Range
Pure CHX solution	28.4 ± 2.1	26.9-29.9	25-32
CHX 3% in Molloplast-B	22.6 ± 1.8	21.3-23.9	20-25
CHX 2% in Molloplast-B	17.4 ± 1.1	16.6-18.2	16-19
CHX 1% in Molloplast-B	13.6 ± 1.1	12.8-14.4	12-15
Pure AgNP suspension	24.2 ± 1.9	22.8-25.6	22-27
AgNP 3% in Molloplast-B	16.0 ± 1.1	15.2-16.8	15-18
AgNP 2% in Molloplast-B	13.2 ± 1.3	12.3-14.1	11-15
AgNP 1% in Molloplast-B	11.4 ± 0.9	10.8-12.0	10-12
Molloplast-B + chloroform	0.0 ± 0.0	–	0
Pure Molloplast-B	0.0 ± 0.0	–	0

1

## Data Availability

The datasets used and/or analyzed during the current study are available from the corresponding author.
